# A rare case of Thoracic Aortic Aneurysm repair post Heart Transplantation

**DOI:** 10.1016/j.jhlto.2024.100183

**Published:** 2024-11-27

**Authors:** Helena Garcia Betinardi Bernardi, Cristhian Espinoza Romero, Vanessa Simioni Faria, Fabiana G. Marcondes-Braga, Fabio A. Gaiotto, Fernando Bacal

**Affiliations:** Heart Institute (InCor), Instituto do Coração, Hospital das Clínicas, HCFMUSP, Universidade de São Paulo, São Paulo, SP, Brazil

**Keywords:** Heart Transplant, Ascending Aortic Aneurysm

## Abstract

During bicaval orthotopic heart transplant (HT), two segments of the aorta are generated, separated by a suture line. Both segments are exposed to the same systemic environment. Nevertheless, it has been observed that aneurysmal disease is confined to one of the segments of aorta. We report here a successful repair of AA, confined to the donor portion of aorta, in a HT recipient after 12 years of transplant.

## Background

During bicaval orthotopic heart transplant (HT), two segments of the aorta are formed, separated by a suture line: one portion from the donor and the other from the recipient.^1^ Despite both segments being exposed to the same systemic environment, aneurysmal disease has been observed to develop exclusively in one segment, without involving the suture line. This results in two distinct types of thoracic aortic aneurysms (TAA)—one in the donor aorta and the other in the recipient's aorta.^1^, ^2^ Patients with these conditions are usually asymptomatic and the disease may not be promptly detected, which can lead to fatal complications. In this report, we present a case of a TAA limited to the donor part of the aorta successfully repaired.

## Clinical Case

A 46-year-old man with advanced heart failure due to Chagas disease with no other comorbidities underwent bicaval orthotopic HT in 2011. The surgery was uneventful, with cross clamp time of 3 hours 50 minutes and cardiopulmonary bypass time of 2 hours 20 minutes. The donor was a 39-year-old man with hypertension whose cause of death was stroke. During early follow up, no acute rejection or significant cardiovascular event were observed. However, multiple bloodstream infections caused by different agents such as *Enterococus faecalis*; *Klebsiella pneumoniae*; *Morganella morganii*; *Acinetobacter baumanni* and *Pseudomonas aeruginosa* occurred and broad-spectrum antibiotics such as Vancomycin, Cefepime and Meropenem were needed. Immunosuppression therapy included cyclosporine and prednisone and no antiproliferative drug was used after 2011 due to leukopenia. During late follow up, the patient developed well-controlled hypertension, dyslipidemia and mild renal insufficiency.

After HT procedure, immediate postoperative echocardiogram showed normal left ventricle ejection fraction and no abnormalities in cardiac valves. The aortic diameter was 34 mm and a mild aortic insufficiency was observed (ratio of the width of the regurgitant jet compared to the diameter of the LV outflow tract < 25% and vena contracta of 0.2 cm).

**Figure 1:**
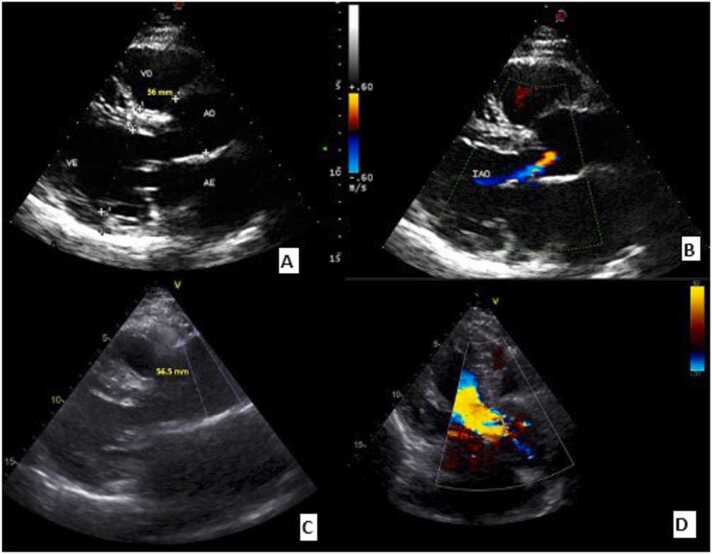
Transthoracic echocardiogram at 2013 (panel A and B) and at 2023 (panel C and D). Panel A depicts the aortic size immediately after heart transplantation (36 mm) and panel B a discrete aortic insufficiency**.** Panel C shows aortic diameter (56.5 mm) and panel D severe aortic insufficiency at the moment of aortic repair.

**Figure 2:**
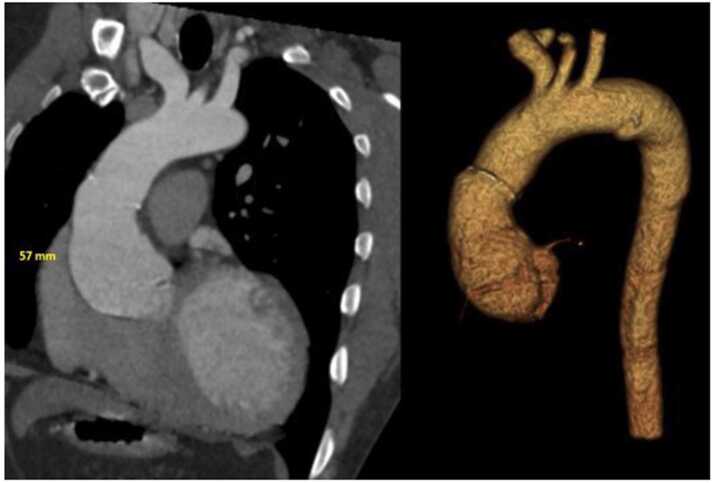
Preoperative aortic computed tomography angiography showing the dilation of the ascending aorta limited to the donor segment of the aorta (not crossing the suture line).

During follow up, a progressive increase in aortic diameter was observed (Figure 1). As of 2022, the aorta growth rate increased and severe dilation of the ascending aorta (57 mm) as well as severe aortic insufficiency were detected (Table 1). The aneurysm was limited to the donor part of the aorta, stopping at the suture line. CT scan confirmed these findings (Figure 2). No left ventricular systolic dysfunction, hypertrophy or cardiac allograft vasculopathy was observed.

**Table 1:** Aortic Dilation by Computed Tomography and Transthoracic Echocardiogram

Ascending Aorta Diameter (mm)						
	2013	2015	2017	2019	2020	2022
CT					45	57
Echo	34	43	44	44	50	56.5
AI grade	discrete	mild	moderate	Moderate	severe	severe

CT: cardiac tomography; Echo; echocardiogram; AI: Aortic insufficiency

Patient underwent a classic Bentall de Bono procedure, as no other surgical option was viable due to significant annular dilation. A size 25 biologic valved conduit was successfully implanted. The aorta tissue showed no changes and the disease was restricted to the donor's heart. The suture line was excised, and the conduit was sutured to the recipient's aorta, with the distal anastomosis completed under clamp. No complication was observed and the patient was discharged after 14 days. The anatomopathological analysis revealed chronic valvopathy with fibrosis and areas of calcification and fusion of commissures.

## Discussion

We report here a successful repair of a thoracic aortic aneurysm, confined to the donor portion of aorta, in a HT recipient after 12 years of transplant. Similar cases have been previously reported. But, most of them involved aneurysms of the recipients’ portion of the aorta. Few cases reported aortic aneurysm confined to the donor portion of the aorta and they are related to the mycotic aortic pseudoaneurysm.^3^

A retrospective review of 296 patients submitted to heart transplant identified 13 patients who developed aortic aneurysm with an average aortic aneurysm growth rate of 1.00 cm/year and an average aortic aneurysm rupture rate of 22.5%/year, which are both significantly higher than in the nontransplant patient population.^4^

Various theories have been proposed to explain aortic aneurysm development after transplant. The most accepted theory suggests that the use of steroids or immunosuppressants may be responsible for aorta diameter increase.^5^ Aortic aneurysm formation is postulated to result from an imbalance between pro and anti-inflammatory vascular endothelial factors. Immunosuppressants may play a role in the development of these post-transplantation aneurysms. Many aortic surgeons feel that steroids are injurious to the aortic wall. A case reported of a kidney transplant patient with a ruptured aortic aneurysm, the histologic evaluation of the aneurysm wall showed complete absence of T cells, B cells, and neutrophils, which were cells found at high levels in a non-immunosuppressed control patient.^6^, ^7^

Also, the mechanical disparities between the two portions of aorta may elevate the risk of developing this condition.^1^, ^2^ Infectious diseases, particularly those caused by fungal agents, may also play a role in the pathophysiology of aortic changes, although this has been less frequently documented. Most post-HT complications are associated with the donor aorta and include aortic rupture, infective pseudoaneurysm of the aorta, and aortic dissection. Finally, genetic predisposition may be considered, as AA are generally confined to one portion of the aorta, leaving the other unaffected despite similar exposure to risk factors and hemodynamic conditions.^1^, ^2^

Regardless of the cause of the development of aortic aneurysms in cardiac transplant patients, their aggressive natural history with high expansion and rupture rates suggests that screening transplant patients for aortic aneurysms may enable early diagnosis and prompt elective repair.

Hence, post-HT follow-up assessments, including echocardiogram focusing on the anastomosis and the two portions of the aorta may be crucial for the heart transplant surveillance.

## Funding

Not applicable.

## CRediT authorship contribution statement

Conceptualization: H.G.B.B. and C.E.R. Methodology: H.G.B.B., C.E.R and F.G.M.B. Investigation: H.G.B.B., V.S.F., C.E.R, F.G.M.B, and F.A.G. Writing—original draft preparation: H.G.B.B. and C.E.R. Writing—review and editing: H.G.B.B., C.E.R, F.G.M.B, F.A.G, F.B. Supervision: F.G.M.B, F.A.G, F.B. All authors have read and agreed to the published version of the manuscript.

## Declaration of Competing Interest

The authors declare that they have no known competing financial interests or personal relationships that could have appeared to influence the work reported in this paper.

## Data Availability

Not applicable.
